# Supplementary material for: Dysregulated pulmonary inflammatory responses exacerbate the outcome of secondary aspergillosis following influenza
Source: mBio. 2023 Sep 8;14(5):e01633-23. doi: 10.1128/mbio.01633-23 (PMC10653922; doi:10.1128/mbio.01633-23)
Supplement: Supplemental material — Supplemental table and figures. [file mbio.01633-23-s0001.docx]

Supplementary Data for Lee et al. Dysregulated Pulmonary Inflammatory Responses Exacerbate the Outcome of Secondary Aspergillosis Following Influenza

Figure 1B:

| Tukey's multiple comparisons test | Summary | Adjusted P Value |
| --- | --- | --- |
| 5 PFU vs. 10 PFU | ns | 0.8774 |
| 5 PFU vs. 25 PFU | ns | 0.8199 |
| 5 PFU vs. 50 PFU | ** | 0.0027 |
| 5 PFU vs. 100 PFU | ** | 0.002 |
| 5 PFU vs. 250 PFU | **** | <0.0001 |
| 5 PFU vs. 1000 PFU | **** | <0.0001 |
| 5 PFU vs. 2500 PFU | **** | <0.0001 |
| 10 PFU vs. 25 PFU | ns | >0.9999 |
| 10 PFU vs. 50 PFU | ns | 0.0789 |
| 10 PFU vs. 100 PFU | ns | 0.0618 |
| 10 PFU vs. 250 PFU | **** | <0.0001 |
| 10 PFU vs. 1000 PFU | **** | <0.0001 |
| 10 PFU vs. 2500 PFU | **** | <0.0001 |
| 25 PFU vs. 50 PFU | ns | 0.1038 |
| 25 PFU vs. 100 PFU | ns | 0.0819 |
| 25 PFU vs. 250 PFU | **** | <0.0001 |
| 25 PFU vs. 1000 PFU | **** | <0.0001 |
| 25 PFU vs. 2500 PFU | **** | <0.0001 |
| 50 PFU vs. 100 PFU | ns | >0.9999 |
| 50 PFU vs. 250 PFU | **** | <0.0001 |
| 50 PFU vs. 1000 PFU | **** | <0.0001 |
| 50 PFU vs. 2500 PFU | *** | 0.0008 |
| 100 PFU vs. 250 PFU | *** | 0.0001 |
| 100 PFU vs. 1000 PFU | **** | <0.0001 |
| 100 PFU vs. 2500 PFU | ** | 0.0011 |
| 250 PFU vs. 1000 PFU | ns | 0.9987 |
| 250 PFU vs. 2500 PFU | ns | >0.9999 |
| 1000 PFU vs. 2500 PFU | ns | 0.9835 |

Figure 1D:

| Paired T test | Summary | Adjusted P Value |
| --- | --- | --- |
| 5x10^6^ *Af* conidia vs. 1x10^7^ *Af* conidia | ** | 0.0019 |

Figure 2C:

| Tukey's multiple comparisons test | Summary | Adjusted P Value |
| --- | --- | --- |
| *Af* only vs. Flu only | **** | <0.0001 |
| *Af* only vs. Flu d0, *Af* at d2 | **** | <0.0001 |
| *Af* only vs. Flu d0, *Af* at d5 | **** | <0.0001 |
| *Af* only vs. Flu d0, *Af* at d8 | **** | <0.0001 |
| *Af* only vs. Flu d0, *Af* at d14 | *** | 0.0007 |
| Flu only vs. Flu d0, *Af* at d2 | *** | 0.0003 |
| Flu only vs. Flu d0, *Af* at d5 | ns | 0.4761 |
| Flu only vs. Flu d0, *Af* at d8 | ns | 0.2665 |
| Flu only vs. Flu d0, *Af* at d14 | ns | 0.794 |

**Supplementary Table 1. Statistical analysis of the weight change curves from Figure 1 and Figure 2.** Weight change curves for the groups shown in Figure 1B and Figure 2C were compared by two-way ANOVA mixed-effects analysis with Tukey’s multiple comparison test. The paired t test was used to compare the two curves shown in Figure 1D.

**
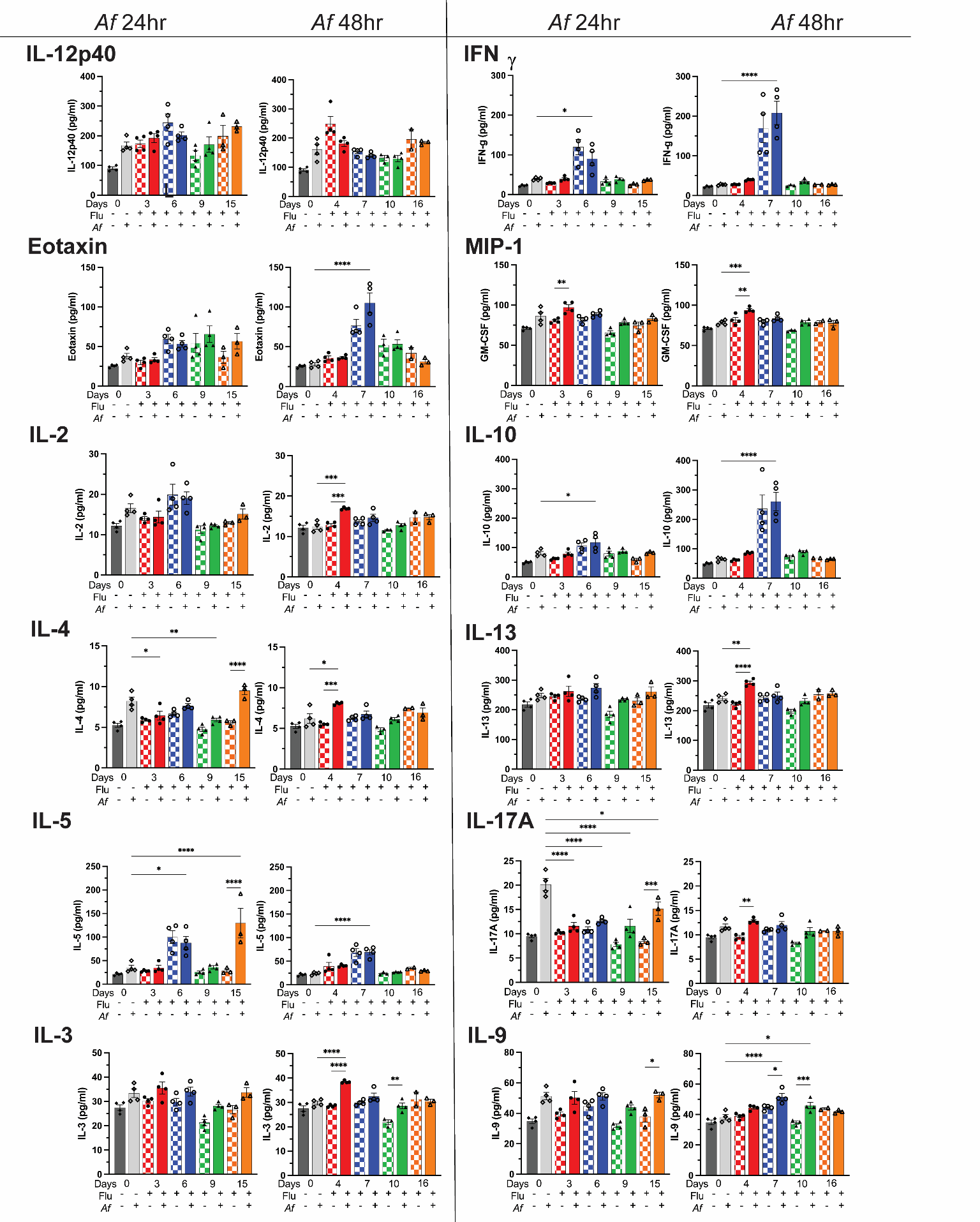
**

**Supplementary Figure S1. Lung cytokine and chemokine concentrations following IAV and *A. fumigatus* single infections and superinfection.** See the Figure 3 legend for details. * P<0.05, ** P<0.005, *** P<0.0005, and **** P<0.0001 by two-way ANOVA with Tukey’s multiple comparison test.

**
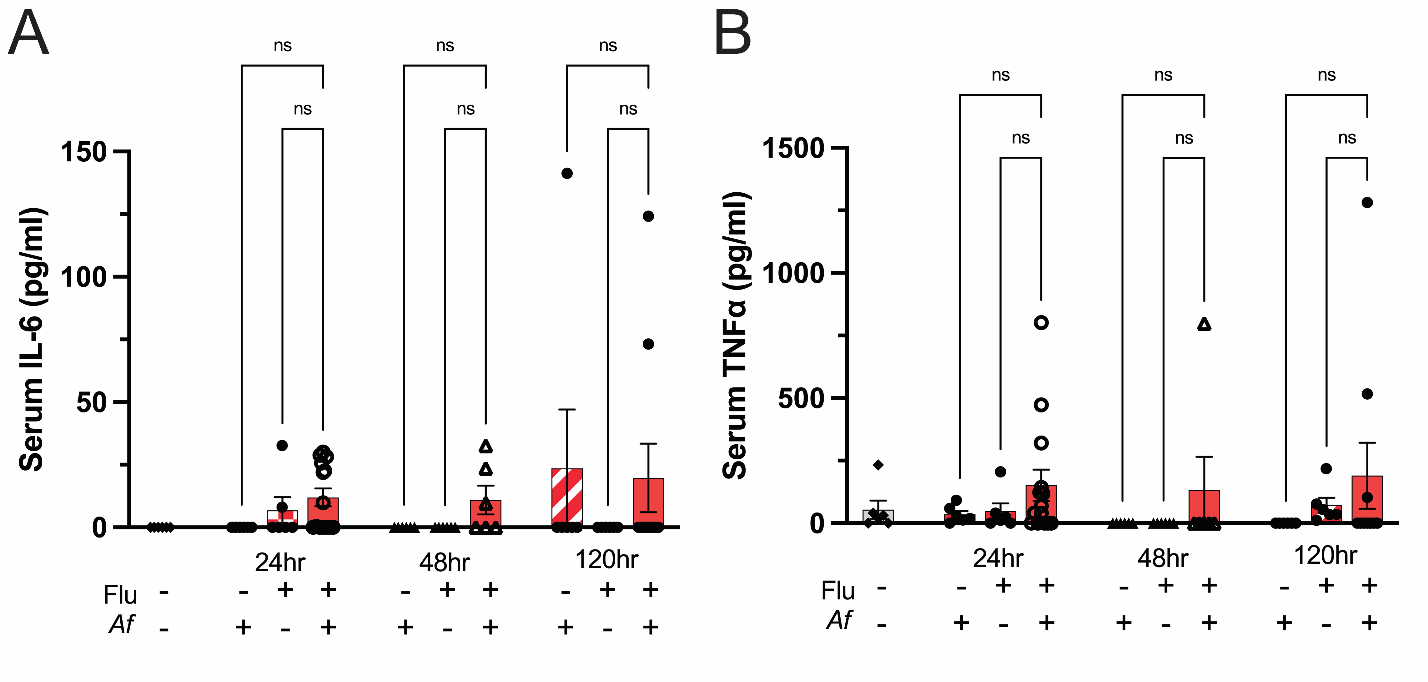
**

**Supplementary Figure S2. IL-6 and TNFα levels in the serum of mice infected with IAV and/or *A. fumigatus*.** Influenza (Flu)-infected mice were challenged with *A. fumigatus* (*Af*) at 2-dpii and serum samples were collected at 24, 48, and 120hr post *A. fumigatus* challenge. Control mice were not infected or singly infected with either influenza or *A. fumigatus*. Expression levels of IL-6 (A) and TNFα (B) were measured by ELISA. The data are combined from 2 independent experiments, each with at least three mice. Each symbol represents an individual mouse. Differences were not significant (ns) when comparing the serum of superinfected mice with the serum of mice that were singly infected with IAV or *A. fumigatus* by two-way ANOVA with Tukey’s multiple comparison test.


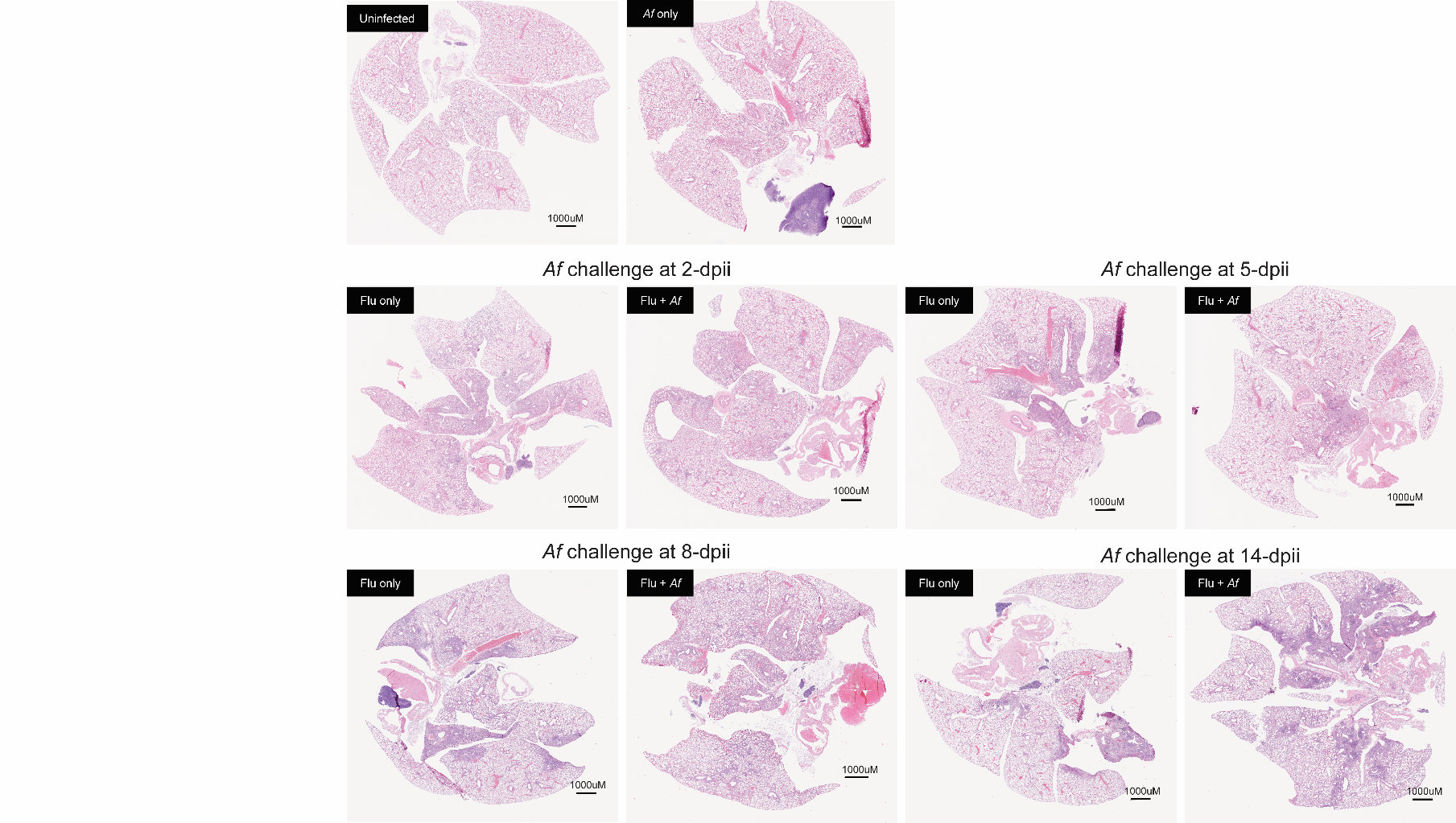


**Supplementary Figure S3. Representative histology of H&E-stained lung samples.** Samples were obtained at 120hr post *A. fumigatus* challenge and scanned at 20X original magnification. Lung samples from the mice that were only infected with IAV were collected at the respective time points of the superinfected mice. Scale bars are 1000 microns.


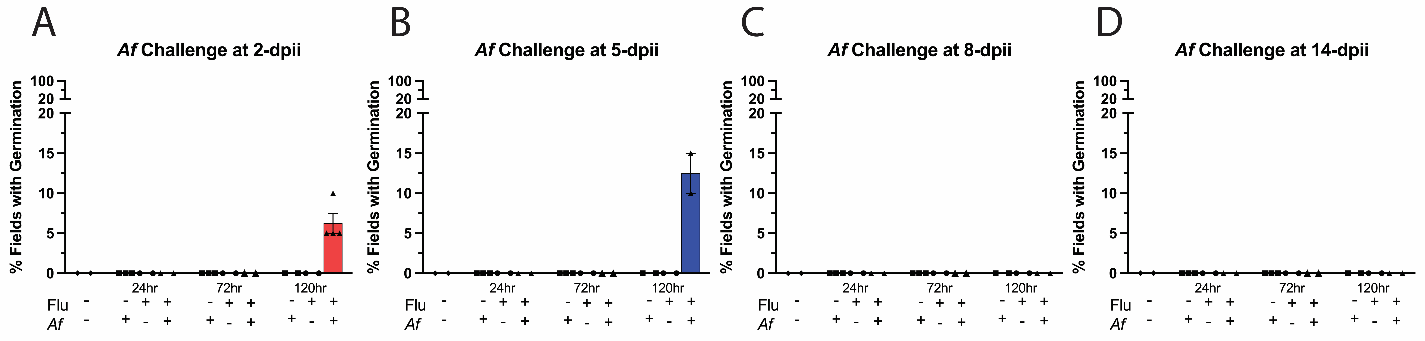


**Supplementary Figure S4. Percentage of fields with germinated conidia.** See Figure 5 legend for details. GMS-stained lung samples from Figure 5A were examined for germinated conidia. Twenty lung fields were randomly selected and scored based on germinated conidia at 20X magnification.


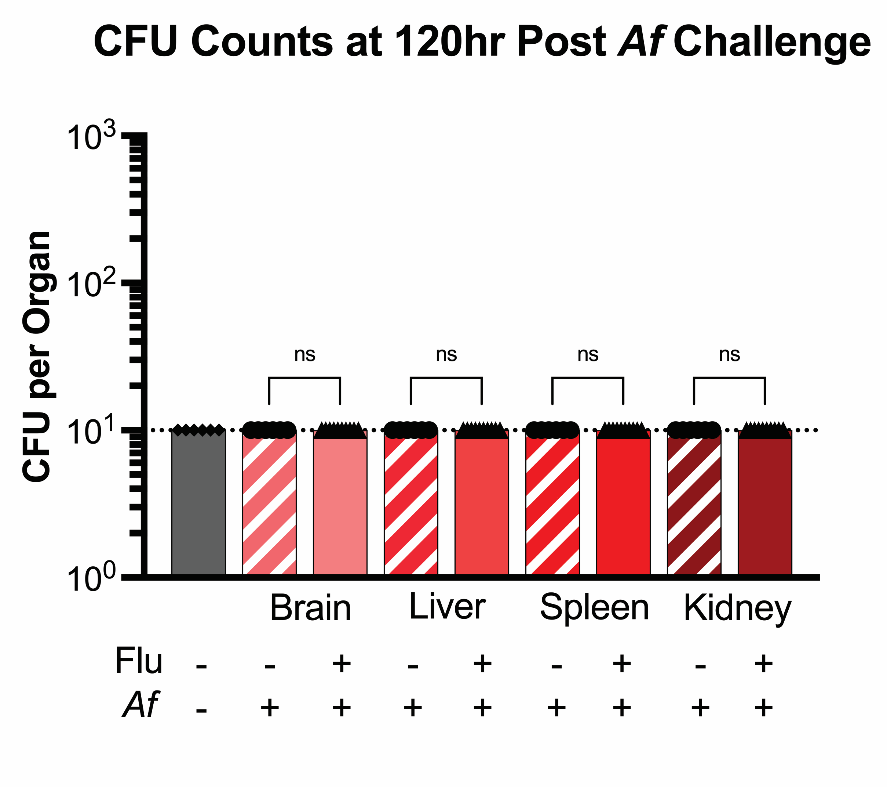


**Supplementary Figure S5. *A. fumigatus* CFU counts from brain, liver, spleen, and kidneys.** Mice were infected with IAV (Flu) and then 2-dpii challenged with *A. fumigatus* (*Af*) as in Figure 6C. Uninfected and singly *A. fumigatus* challenged mice were used as controls. Organ samples were collected 120hr after *A. fumigatus* challenge. The data shown are the combination of two independent experiments, each with at least three mice/group. The data are expressed as means ± SEM. Each symbol represents an individual mouse. Differences were not significant (ns) when comparing *A. fumigatus* only challenged mice with superinfected mice by two-way ANOVA with Tukey’s multiple comparison test. Dotted line represents the lower limit of detection (LLD) of the assay. Numbers at or below the LLD were assigned the value of the LLD.


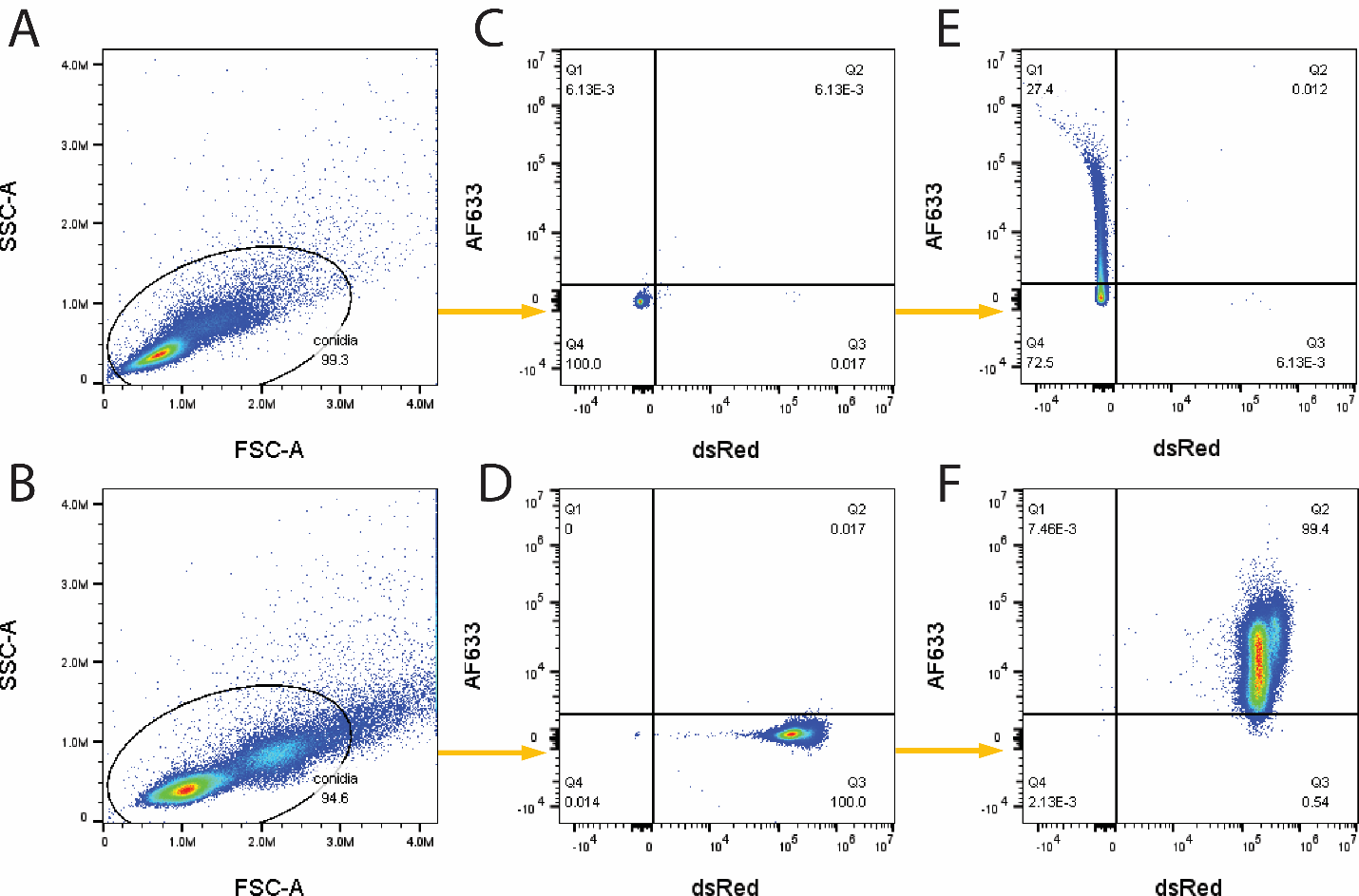


**Supplementary Figure S6. Flow cytometry analysis of FLARE-stained conidia.** *A. fumigatus* CEA10 and dsRed conidia were gated to exclude debris and cell clumps (A and B). The conidia population was then plotted against Alexa Fluor 633 and dsRed. (C) CEA10 conidia, which served as a negative control for dsRed staining. (D) dsRed conidia, which are genetically encoded to express dsRed. The conidia were then secondarily stained with the fluorophore Alexa Fluor 633. The control CEA10 conidia (E) show staining with just Alexa Fuor 633 whereas the FLARE conidia stain with both Alexa Fluor 633 and dsRed (F).


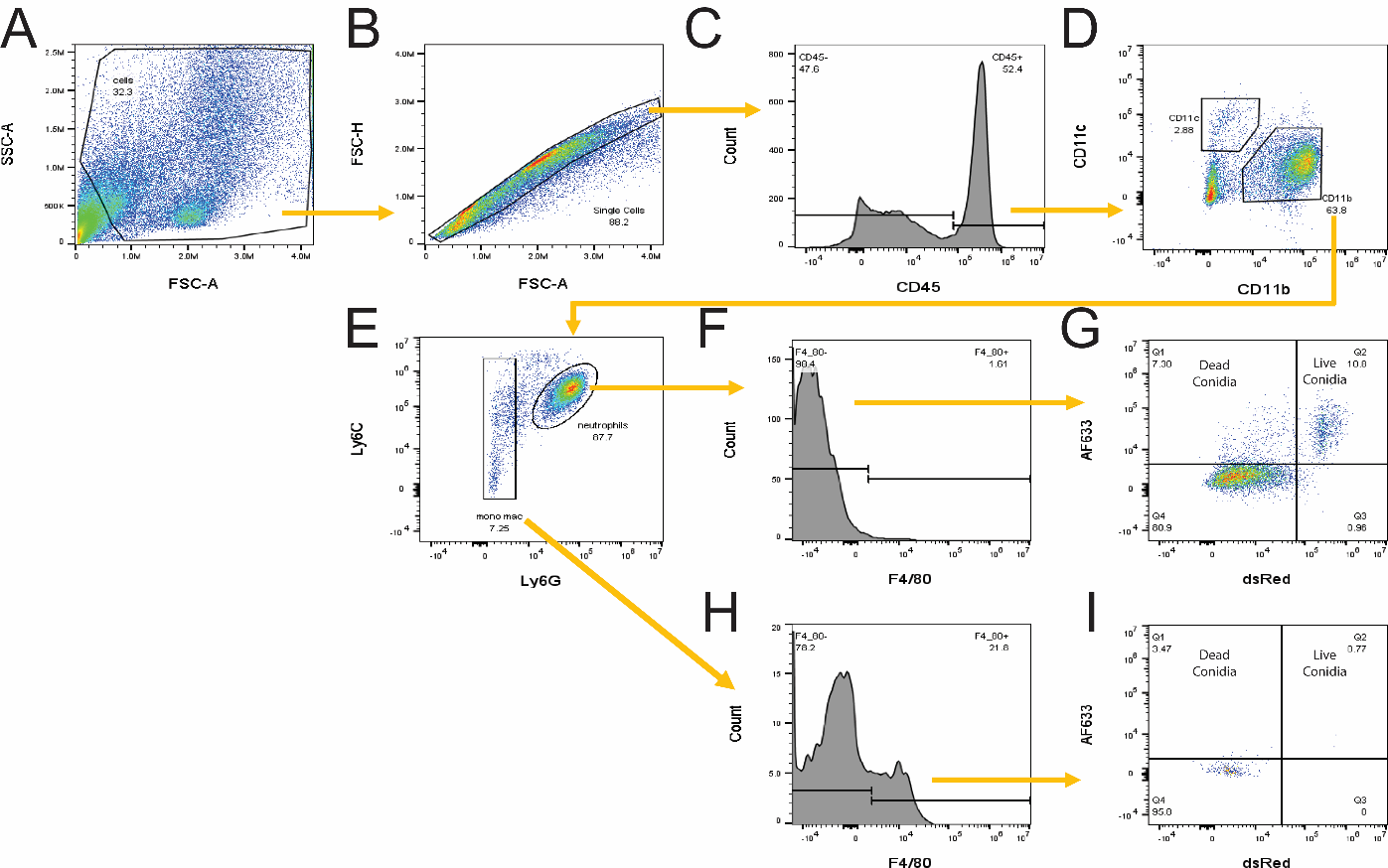


**Supplementary Figure S7. Gating strategy for flow cytometry analysis.** In the example shown, lung cells were analyzed 1 days following infection with 3 x 10^7^ *A. fumigatus* FLARE conidia. (A) Debris was gated out from the cell population. (B) The single cell population was gated. (C) Leukocytes were selected from the single cell population by gating on the CD45^+^ population. (D) The leukocytes were separated into CD11c^+^ and CD11b^+^ cell populations. (E) The CD11b^+^ cell population was gated for monocytes/macrophages (mono/macs) (Ly6C^+/-^, Ly6G^-^) and neutrophils (Ly6C^+^, Ly6G^+^). (F) Neutrophils were further separated by gating on F4/80^-^. (G) To analyze uptake and killing of FLARE conidia, the neutrophils were gated for Alexa Fluor 633 and dsRed. Neutrophils containing live FLARE conidia are dsRed^+^ and Alexa Fluor 633^+^, whereas neutrophils containing only dead FLARE conidia are dsRed^-^ and Alexa Fluor 633^+^. Neutrophils without conidia are dsRed^-^ and Alexa Fluor 633^-^ and appear in Q4. (H) Macrophages were distinguished from monocytes by selecting for F4/80^+^ cells from the Ly6C^+/-^, Ly6G^-^ population. (I) dsRed and Alexa Fluor 633 population were gated on the macrophages to examine FLARE conidia uptake and killing using the same strategy described for neutrophils.


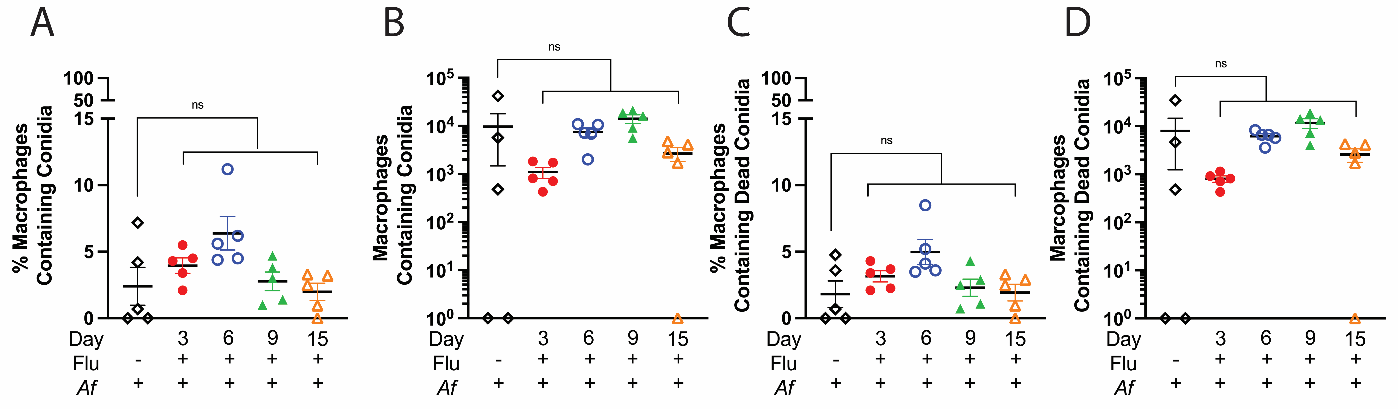


**Supplementary figure S8. Phagocytosis and killing of *A. fumigatus* FLARE conidia by macrophages.** The experimental design was the same as in Figure 8 except the macrophage population in the lungs was examined. (A) and (B) The percentage of macrophages containing conidia and the total number of macrophages with conidia, respectively. (C) and (D) Percentage of macrophages and total number of macrophages containing only dead conidia, respectively. Comparisons of the superinfected groups with the mice singly challenged with *A. fumigatus* were not significant (ns) by one way ANOVA. Data are the combination of ≥5 independent experiments at different time points and each symbol represents an individual mouse.


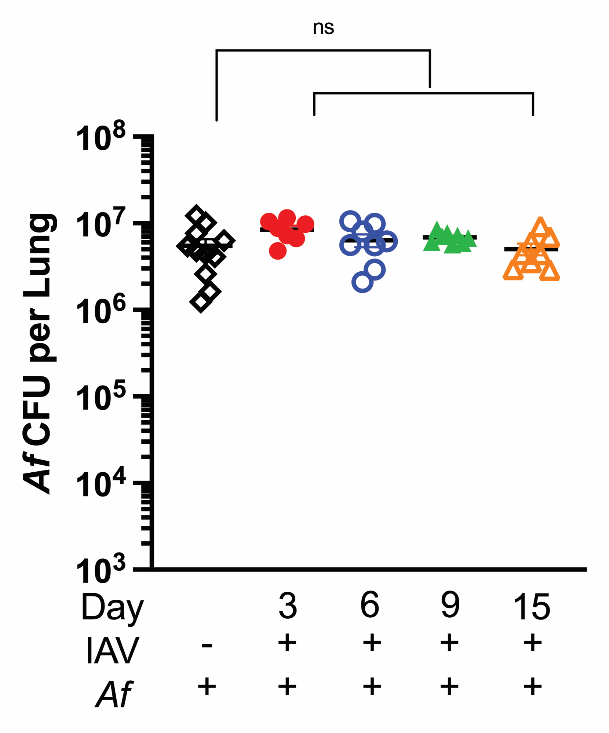


**Supplementary figure S9. CFU counts from the FLARE conidia assay**. See Figure 7 for experimental details. Ten µl were taken from each of the lung samples for CFU plating. No significant (ns) differences were seen when comparing the mice infected with just *A. fumigatus* FLARE conidia and mice dually infected by one way ANOVA. Data are the combination of ≥5 independent experiments at different time point and each symbol represents an individual mouse.
